# Vaginal pH measured in vivo: lactobacilli determine pH and lactic acid concentration

**DOI:** 10.1186/s12866-019-1388-8

**Published:** 2019-01-14

**Authors:** Deirdre Elizabeth O’Hanlon, Richard A. Come, Thomas R. Moench

**Affiliations:** 1 Institute for Genomic Sciences, University of Maryland School of Medicine, 801 West Baltimore Street, Baltimore, MD USA; 2 Thomas C. Jenkins Department of Biophysics, Johns Hopkins University, Baltimore, Maryland USA; 3 ReProtect Inc., Baltimore, Maryland USA

**Keywords:** Lactobacilli, Microbiota, Vaginal, Lactic acid, pH, Bacteria, Bacterial vaginosis

## Abstract

**Background:**

Lactic acid (protonated lactate) has broad antimicrobial activity. Vaginal lactobacilli produce lactic acid, and are known to confer protection against reproductive tract infections when they are predominant in the vaginal microbiota. Using novel ex vivo methods, we showed that cervicovaginal fluid (CVF) from women with a predominantly lactobacilli-morphotype microbiota contains significantly more lactic acid than previously thought, sufficient to inactivate reproductive tract pathogens.

Here, we measured vaginal pH in vivo in 20 women with a predominantly lactobacilli-morphotype (low Nugent score) microbiota. We also investigated the in vitro production of protons (as hydrogen ions) and lactate by vaginal lactobacilli.

**Results:**

The average vaginal pH in these women was 3.80 ± 0.20, and the average lactate concentration was 0.79% ± 0.22% *w*/*v*, with pH and lactate concentration tightly correlated for each sample. In vitro, lactobacilli cultured from these CVF samples reached an average pH of 3.92 ± 0.22, but the average lactate concentration was only 0.14% ± 0.06% *w*/*v*, approximately five-fold less than in the corresponding CVF samples. When the pH of the cultures was raised, lactate and hydrogen ion production resumed, indicating that production of lactate and hydrogen ions by vaginal lactobacilli is limited primarily by their sensitivity to hydrogen ion concentration (low pH) not lactate concentration.

**Conclusions:**

Some vaginal lactobacilli cultures have a lower limiting pH than others, and limiting pHs in vitro showed good correlation with pHs measured in vivo*.* The limiting pH of the lactobacilli predominant in a woman’s vaginal microbiota seems critical in determining the concentration of antimicrobial lactic acid protecting her.

## Background

The composition of the vaginal microbiota is known to alter dramatically a woman’s resistance or susceptibility to reproductive tract infections. Women with a low Nugent score (predominantly lactobacilli-morphotype) microbiota are at reduced risk of most sexually transmitted infections including HIV-1 [1, 2], gonorrhea [3, 4] and trichomoniasis [5, 6], as well as obstetric infections [7, 8] that contribute to preterm deliveries and perinatal complications, infection by bacteria implicated in pelvic inflammatory disease [9, 10] and other gynecologic infections [11, 12], and urinary tract infections [13, 14], indicating that vaginal lactobacilli have broad antimicrobial activity against viral, bacterial, and eukaryotic pathogens in the reproductive tract.

Lactobacilli have been shown to disrupt infectious processes in many ways, including inhibiting pathogen growth [15, 16], adherence to host cells [17, 18] and formation of biofilms [19, 20], as well as modulation of cytokine [21, 22] and receptor [23, 24] expression by host cells. The role of lactic acid production by vaginal lactobacilli is relatively unstudied: lactic acid production is ubiquitous among all species and strains of lactobacilli and does not, therefore, appear useful in distinguishing between maximally and minimally protective isolates. Additionally, some attempts to demonstrate significant lactic acid mediated inactivation of pathogens by lactobacilli in vitro were unsuccessful [25, 26], leading to a general assumption that lactic acid contributes only minor antimicrobial activity in vivo.

In a prior study [27], we used novel ex vivo methods of sampling and analyzing cervicovaginal fluid (CVF), obviating the usual dilution and loss of physiological carbon dioxide (CO_2_). Using these ex vivo methods, we showed that the average lactic acid concentration in CVF from 56 women with a predominantly lactobacilli-morphotype microbiota was approximately 11-fold higher than previously thought ‘normal’. We have previously shown that this new, higher concentration of lactic acid is more than sufficient to inactivate almost all species of bacteria associated with bacterial vaginosis [28]; other investigators have shown it is also ample to inactivate HIV-1 [29], HSV-1 and HSV-2 [30], *Chlamydia trachomatis* [31], and *Neisseria gonorrhoeae* [32].

In this study, we made direct, in vivo measurements of vaginal pH in 20 women with a predominantly lactobacilli-morphotype microbiota, to further validate our earlier ex vivo findings. We also examined the relationship between the concentration of lactic acid in CVF samples and the concentration of lactic acid produced in vitro by lactobacilli cultured from the samples, both to understand why lactic acid mediated inactivation by lactobacilli was ineffective in vitro and – more importantly – to begin understanding the factors that determine how much protective lactic acid is present vaginally.

## Results

A total of 22 participants were recruited; they were between 19 and 37 years old (mean age 26 ± 5 years), and self-identified as non-Hispanic white (*n* = 17), Black (*n* = 2), or Asian (*n* = 3). One CVF sample was excluded due to Nugent score > 3, and one participant was excluded due to incomplete in vivo data collection. Data from the remaining 20 participants and their CVF samples are presented here.

### Vaginal pH and lactate

**Fig. 1:**
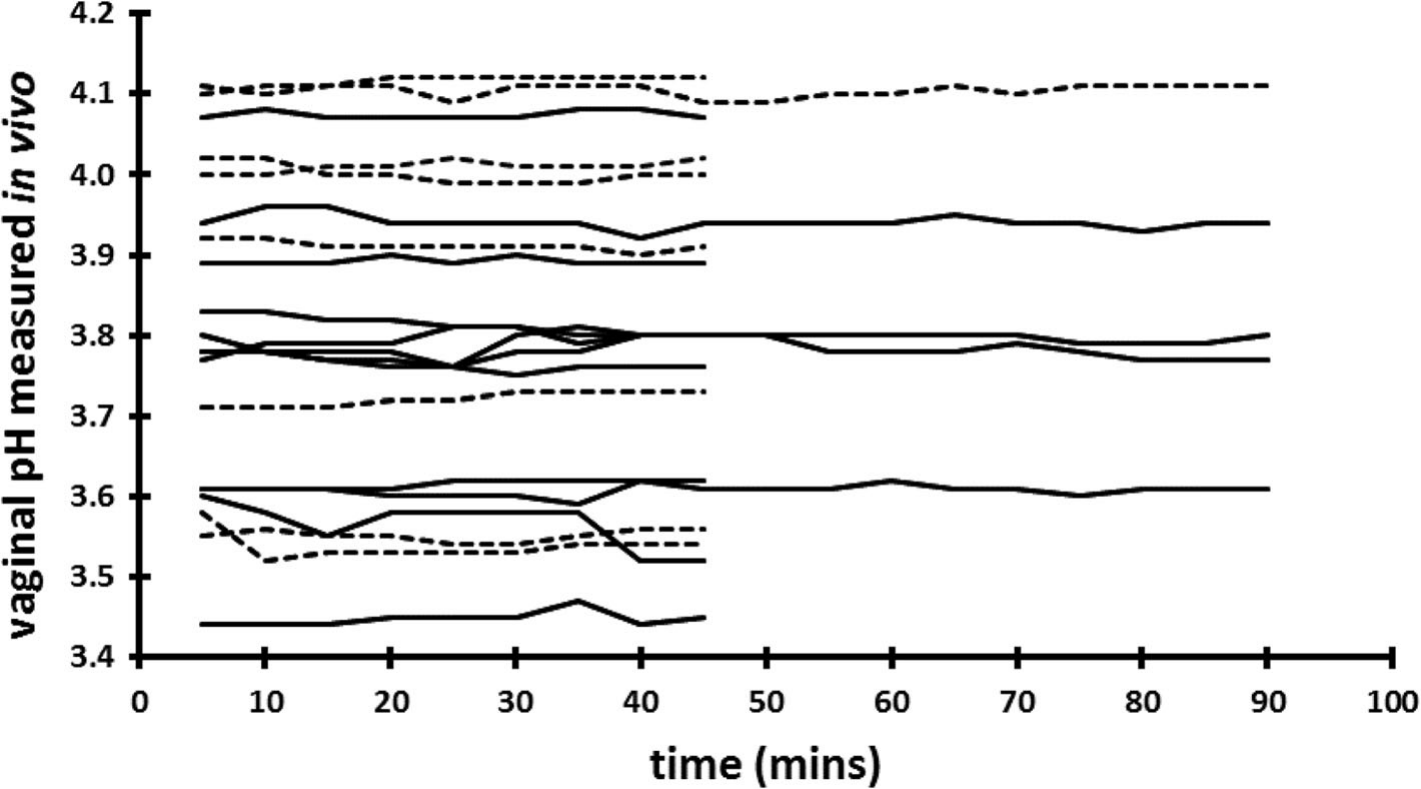
Vaginal pH measured in vivo in 20 women with Nugent score 0–3, showing stability of recorded values over time. Dashed lines represent women whose cervicovaginal fluid (CVF) contained only L-lactic acid; solid lines represent women whose CVF contained both D- and L-lactic acid

**Fig. 2:**
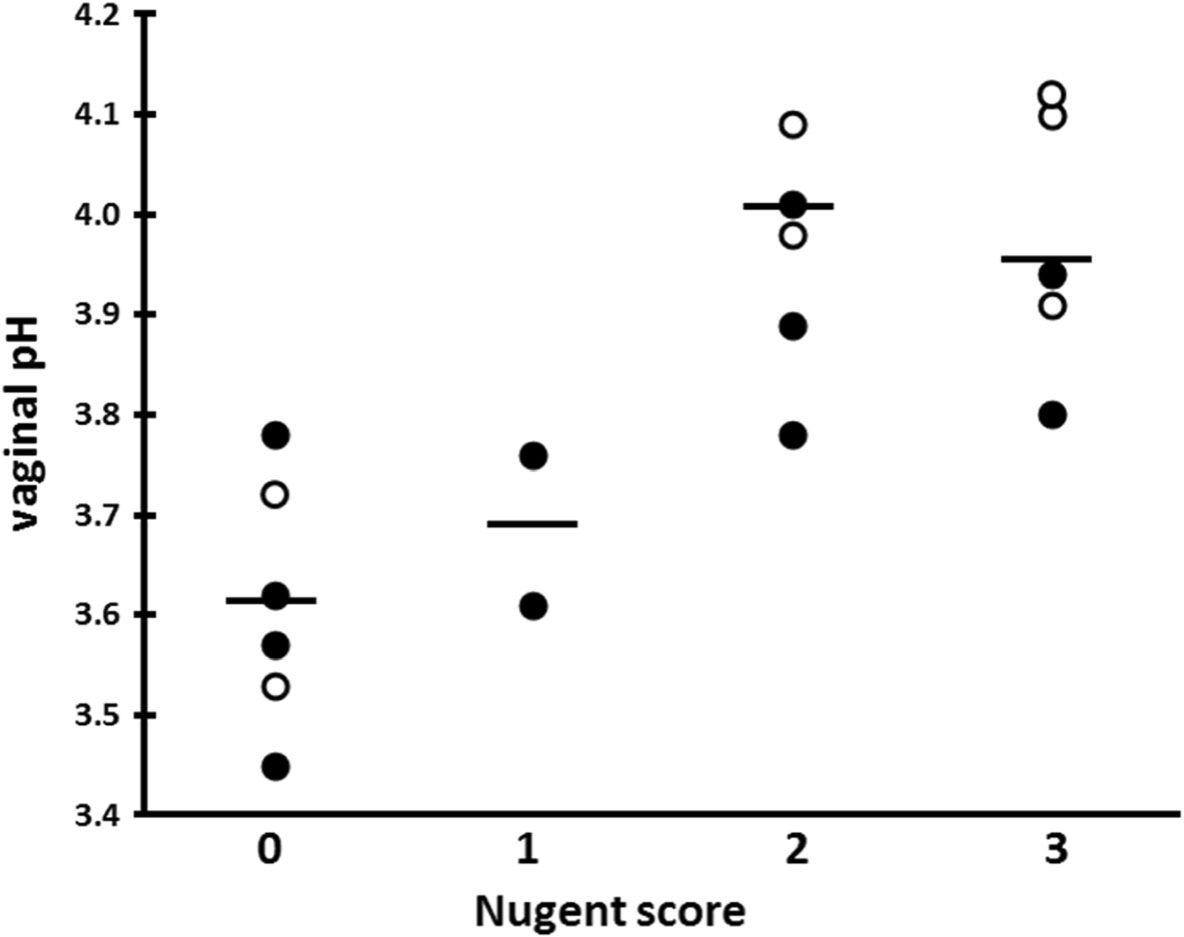
Vaginal pH measured in vivo, grouped by Nugent score. Open circles represent women whose CVF contained only L-lactic acid; filled circles represent women whose CVF contained both D- and L-lactic acid

We have previously shown [27] that the hypoxic (low oxygen) and hypercapnic (high carbon dioxide) condition of the vagina contributes to the acidity of CVF; other investigators have demonstrated that vaginal insertion of a diaphragm [33] or tampon [34] temporally alters the partial pressure of gases inside the vagina. Vaginal insertion of the #A57184 electrode does not involve as much distention of the vulva as diaphragm insertion, nor does the #A57184 contain a bolus of air as a tampon does. We therefore reasoned that insertion of the electrode would minimally perturb the gas content of the vagina. To be certain, we began by measuring the vaginal pH for 90 min after electrode insertion to ensure any perturbation had resolved. In fact, as Fig. 1 shows, most measurements stabilized within 5 to 10 min of electrode insertion; subsequent measurements were made for a period of 45 min only. The mean vaginal pH measured in vivo was 3.80 ± 0.22 (range 3.45 to 4.12). We found that participants with Nugent score 0 and 1 had significantly lower vaginal pHs (mean 3.62 ± 0.11) compared to participants with Nugent score 2 and 3 (3.95 ± 0.12) (Fig. 2). This is consistent with the lower mean vaginal pH observed in our earlier study [27], in which approximately 80% of participants had a Nugent score 0 or 1, compared to the more disparate distribution in this study.

**Fig. 3:**
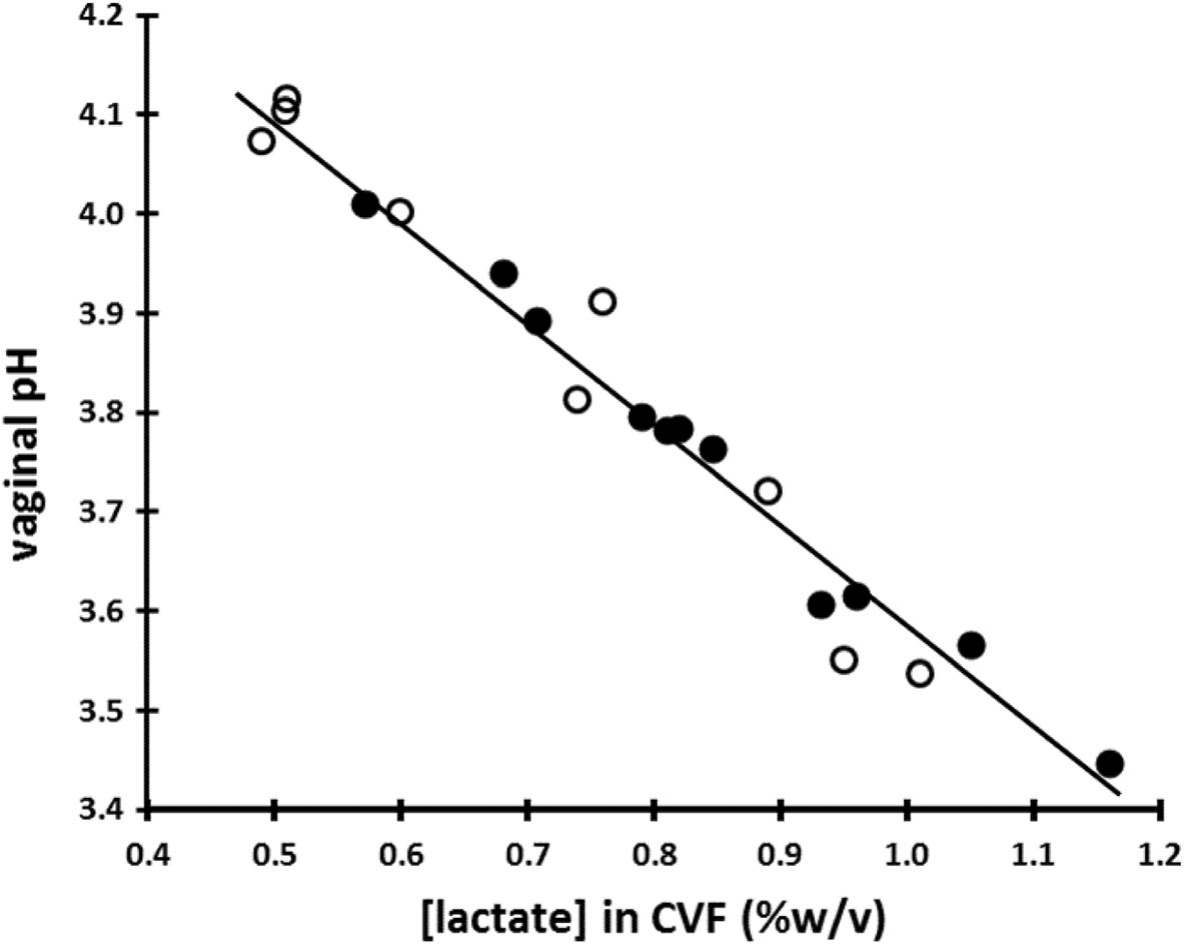
In 20 women with Nugent score 0–3, the vaginal pH measured in vivo correlates very tightly (*r*^*2*^ = 0.97) with the lactate concentration of the ex vivo CVF. Open circles represent women whose CVF contained only L-lactic acid; filled circles represent women whose CVF contained both D- and L-lactic acid

**Fig. 4:**
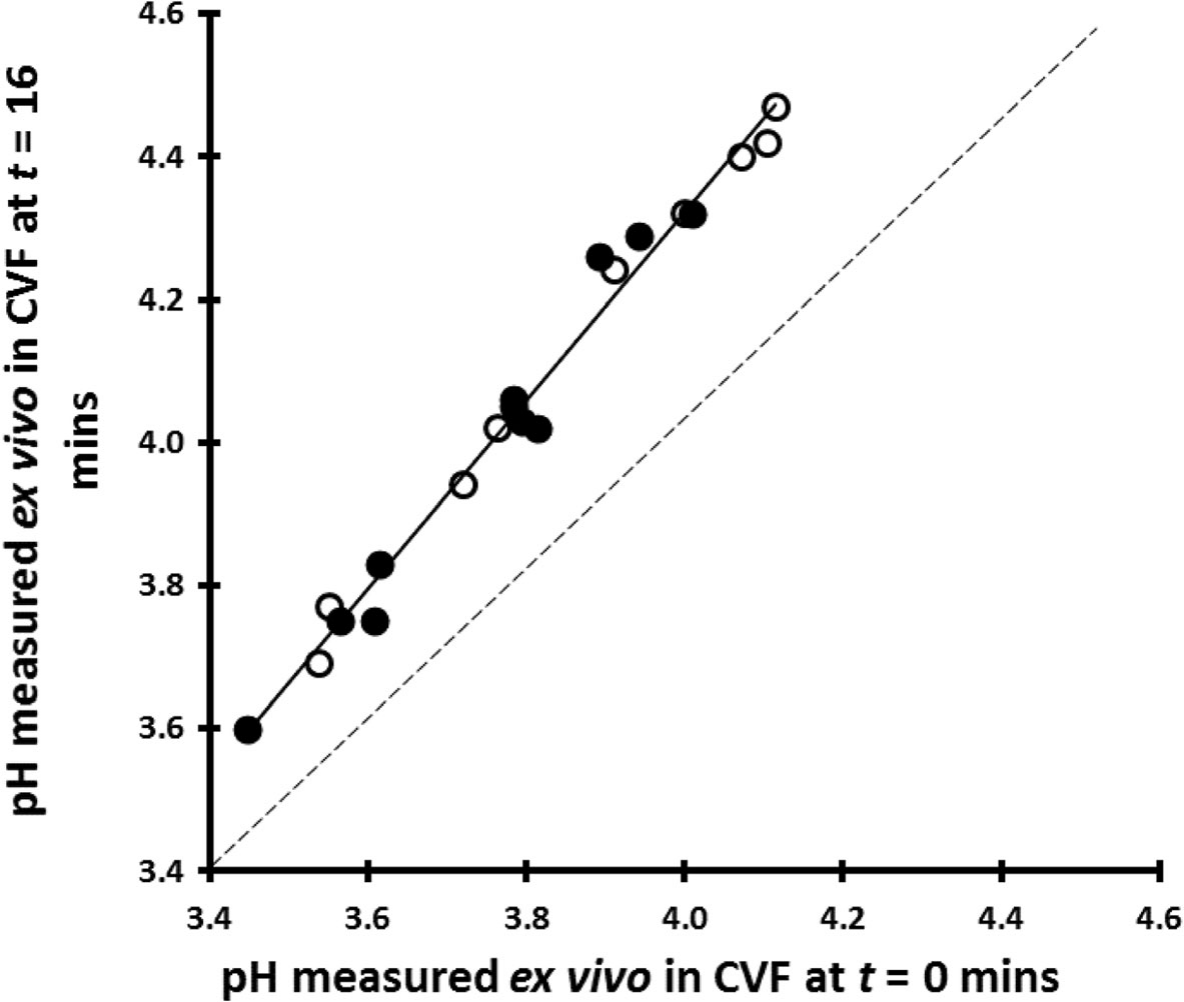
Increase in pH of 20 cervicovaginal fluid samples, probably due to loss of physiological CO_2_, showing that the increase is less in samples with lower starting values. Open circles represent women whose CVF contained only L-lactic acid; filled circles represent women whose CVF contained both D- and L-lactic acid

The measurement of pH ex vivo (CVF freshly collected on an Instead Softcup) confirmed our previous finding [27] that the pH of CVF exposed to air increases by approximately 0.3 pH units over a period of about 2 min, presumably due to loss of physiological CO_2_. The mean lactate concentration in these 20 samples was 0.79% ± 0.20% *w*/*v* (range 0.49 to 1.16% *w*/*v*); we found a tight inverse correlation (*r*^*2*^ = 0.97) between vaginal pH and lactate concentration (Fig. 3). We also observed that CVF samples with lower starting pHs (and hence more buffering by lactate) showed less increase in pH over time than CVF samples with higher starting pH values (Fig. 4).

### Hydrogen ion and lactate production by vaginal lactobacilli in vitro

**Fig. 5:**
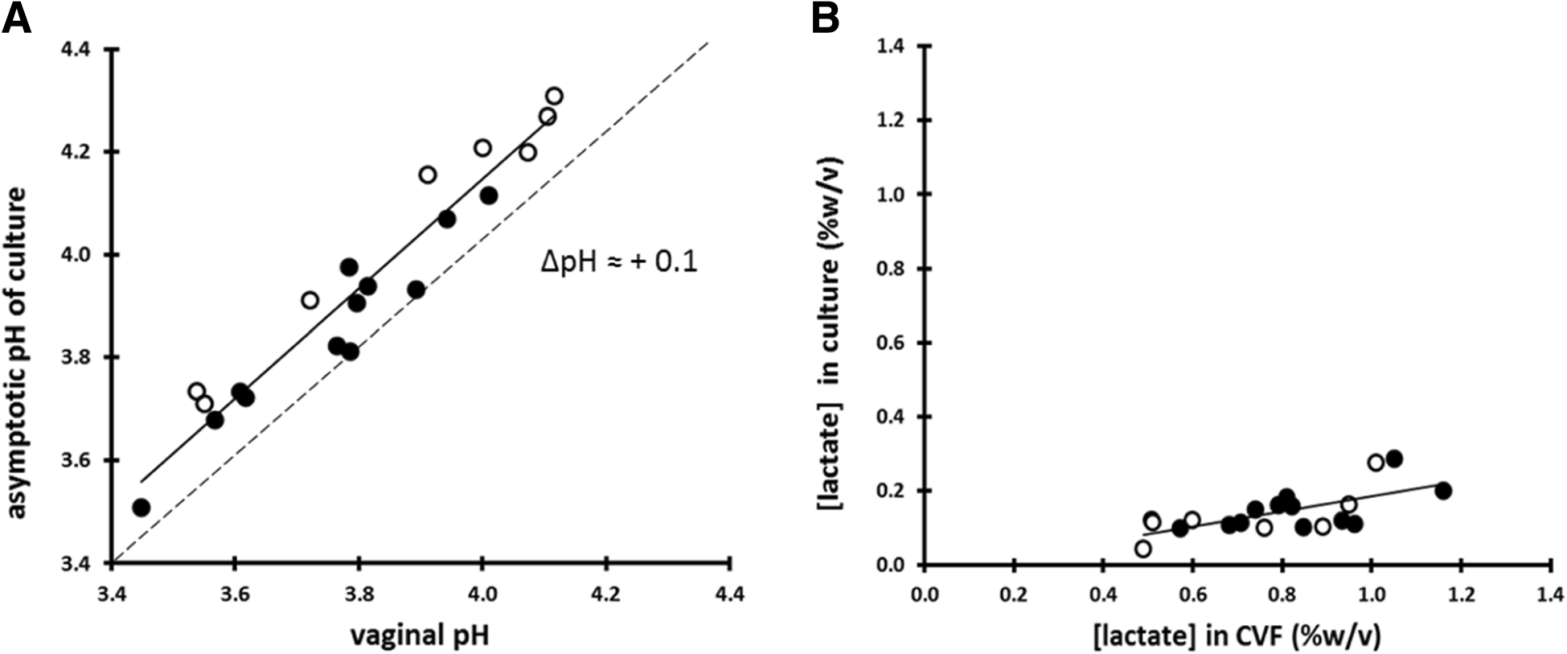
**a** The asymptotic pH achieved in vitro by lactobacilli is highly correlated (*r*^*2*^ = 0.93) with the vaginal pH of the woman from which the lactobacilli were isolated. Open circles represent women whose CVF contained only L-lactic acid; filled circles represent women whose CVF contained both D- and L-lactic acid. **b** The concentration of lactate in the supernatants from the in vitro culture correlates more loosely (*r*^*2*^ = 0.44) with the lactate content of the CVF samples from which the lactobacilli were isolated. Open circles represent women whose CVF contained only L-lactic acid; filled circles represent women whose CVF contained both D- and L-lactic acid

The asymptotic pH attained by each in vitro lactobacilli culture correlated well (*r*^*2*^ = 0.93) with the corresponding in vivo pH measurement, although the in vitro pH was, on average, 0.13 units higher than the in vivo pH (Fig. 5a). Eight cultures contained only L-lactic acid, and the remaining 12 cultures contained both D- and L-lactic acid; there was a perfect correlation between the D:L ratio of each culture and the D:L ratio of the corresponding CVF sample from which the lactobacilli were cultured (data not shown). However, the mean *concentration* of lactate in the supernatants from the in vitro cultures was only 0.14% ± 0.06% *w*/*v* (range 0.10 to 0.29% *w*/*v*), approximately one-quarter that of the CVF samples (Fig. 5b). In all cases, the elevation of the pH by the addition of sodium hydroxide (NaOH) prompted further production of lactate and hydrogen ions (Table 1).

**Table 1:** pH and lactate concentration of whole-sample lactobacilli cultures at ^1^asymptote (after 2–5 days of incubation) and ^2^after neutralization and 2 additional days of incubation. (Samples were numbered in order of ascending pH^1^ for this table only)

Sample #	pH^1^	Lactic acid (%w/v)^1^	pH^2^	Lactic acid (%w/v)^2^
01	3.51	0.32	3.51	0.45
02	3.68	0.31	3.67	0.40
03	3.70	0.29	3.69	0.38
04	3.71	0.20	3.72	0.28
05	3.72	0.17	3.72	0.24
06	3.73	0.18	3.74	0.22
07	3.80	0.18	3.81	0.26
08	3.81	0.20	3.80	0.31
09	3.90	0.15	3.90	0.20
10	3.91	0.16	3.90	0.21
11	3.92	0.13	3.91	0.18
12	3.93	0.12	3.94	0.18
13	3.97	0.11	3.98	0.15
14	4.08	0.11	4.09	0.17
15	4.10	0.10	4.08	0.14
16	4.15	0.09	4.14	0.13
17	4.20	0.09	4.20	0.14
18	4.21	0.08	4.21	0.16
19	4.29	0.05	4.30	0.11
20	4.32	0.05	4.30	0.14

## Discussion

Protonated lactic acid, rather than deprotonated lactate anion, is known to be the active, microbicidal form [28, 29]; lactic acid concentration increases with both increasing lactate concentration and increasing hydrogen ion concentration (*decreasing* pH). Lactic acid concentration is calculated using the Henderson-Hasselbach equation and the *p*K_*a*_ of lactic acid (3.86). An extensive review [35] of published measurements found the mean observed lactate concentration to be 0.2% (22 mM) and pH to 4.2, yielding a lactic acid concentration of 0.06% (7 mM). These values are commonly accepted as ‘normal’, though most of the studies reviewed included no assessment of microbiotal health and used relatively imprecise pH papers (discussed in [27]). Our finding of 0.79% and pH 3.90 yields a lactic acid concentration of 0.42% (47 mM), seven-fold higher. Lactic acid at 0.06% has little to no effect on reproductive tract pathogens, whereas at 0.42% it potently inactivates BV-associated bacteria [28], HIV-1 [29], HSV-1 and HSV-2 [30], *C. trachomatis* [31], and *N. gonorrhoeae* [32].

We observed that in vivo and in vitro asymptotic pHs correlated much more tightly than in vivo and in vitro lactate concentrations, and that lactate production resumed after elevating the pH with NaOH. This suggests that production of lactate and hydrogen ions by vaginal lactobacilli is primarily limited by sensitivity to hydrogen ion concentration (low pH). In vitro, lactobacilli are hampered by the limited pH-buffering capacity of growth medium in a non-permeable container, compared to the constant renewal of buffering capacity in vivo through production of host proteins, lipids, etc., and biophysical alleviation as lactic acid diffuses across the cervicovaginal epithelium.

Some lactobacilli cultures had a lower in vitro asymptotic pH than others, corresponding with lower in vivo pHs, further supporting the hypothesis that it is the low-pH tolerance of the lactobacilli in the microbiota that determines vaginal pH (rather than variations in individual epithelial permeability, epithelial metabolism, or other factors).

*Lactobacillus iners* is unique among vaginal lactobacilli in producing only L-lactic acid [36]. We can tentatively identify the eight CVF samples containing only L-lactic acid as predominated by *L. iners*. Predominance of *L. iners* has been associated with lower vaginal lactic acid concentration and higher vaginal pH [37, 38], but we found no association between the presence of only the L-isomer and the total concentration of lactic acid or the pH. This observation suggests that at least some strains of *L. iners* are capable of producing high lactic acid and low pH, supporting the hypothesis that some *L. iners* can be protective [39].

## Conclusions

As described at the beginning of this report, vaginal lactobacilli are believed to protect against reproductive tract pathogens via multiple activities, and studies have distinguished Lactobacillus spp. and strains based on their degree of activity. Production of lactic acid by vaginal lactobacilli is relatively overlooked, possibly because in vitro production is inadequate for pathogen inactivation. In this study, however, we find that in women with a predominantly lactobacilli-morphotype microbiota, in vivo production of lactic acid is much higher and more than sufficient for the inactivation of most reproductive tract pathogens. Furthermore, we find that it is primarily the pH-tolerance of each women’s vaginal lactobacilli that determines her vaginal pH.

To further elucidate the role of the production of lactic acid by vaginal lactobacilli, we are currently investigating the in vivo production capacity of lactobacilli cultured from women who do not have a predominantly lactobacilli-morphotype microbiota. We are also undertaking a longitudinal study of lactic acid production by vaginal lactobacilli in women whose microbiota undergoes compositional changes.

## Methods

### Reagents

Unless otherwise stated all reagents were supplied by Sigma-Aldrich Inc. (St. Louis, MO).

### Study participants

The study was carried out at the Johns Hopkins University campus, between October 2015 and March 2016. Study participants were recruited from among students and staff at the university and neighboring campuses. Study participants were required to be 18–45 years old, in good general health, at least 3 days past most recent menses and unprotected penile-vaginal intercourse, at least 3 weeks past most recent use of systemic or vaginal antibiotics and antifungals, and free of vaginal symptoms (itching, odor, discharge or discomfort).

### Ethics, consent and permissions

Each study participant gave written informed consent under protocol NA_00083620, approved by the Johns Hopkins Medicine Institutional Review Board on Human Subject Research.

### Measurement of vaginal pH in vivo

Vaginal pH was measured in vivo using the #A57184 combination Ag/AgCl electrode (Beckman Coulter Inc., Sharon Hill PA); this is a sealed, epoxy-bodied electrode comparative in texture and dimensions to a plastic tampon applicator. The electrode was connected to an Orion Star A221, battery-powered portable pH meter (Thermo Fisher Scientific, Waltham MA).

A new electrode was prepared for each study participant immediately before use: under aseptic conditions, the electrode was removed from the manufacturer’s packaging and calibrated in fresh standard solution pH 7.00, 4.00, and 2.00, which had been passed through 0.20 μm sterile syringe filters (Corning Inc., Corning NY). The dH_2_O used to rinse the electrode during and after calibration was previously sterilized by autoclaving, and UV-treated to guard against phage contamination. The prepared electrode was placed upright in a sterile 50 mL polypropylene conical tube (Corning Inc.) and given to the study participant.

Each study participant partially undressed and, reclining, vaginally inserted the electrode and remained in the supine position. The participant used the pH meter’s single-button ‘measure’ function to record the pH immediately after electrode insertion and then at approximately 5-min intervals for total periods or 45 or 90 min. The pH values and the exact time of measurement were automatically recorded by the ‘data log’ function of the pH meter.

### Measurement of vaginal pH ex vivo in air

We wished to compare the measurements made in vivo with our previously reported [27] ex vivo measurements; to facilitate this comparison we also made ex vivo measurements for the participants in this study. At the end of the in vivo measurements, the participant removed the electrode from her vagina and immediately collected a sample of her CVF using an Instead® Softcup™. We have previously described [27, 40] how the use of this non-absorbent, disposable menstrual device permits collection of relatively large, minimally perturbed CVF samples. The participant vaginally inserted the Softcup, removed it, and placed it in a sterile 100 mm polystyrene petri dish (Thermo Fisher Scientific). The investigator quickly placed the electrode so that its junction made positive contact with the layer of CVF on the Softcup; a clamp-stand was used to hold the electrode in place. The first ex vivo measurement was made as soon as the electrode was positioned on the Softcup (≤30 s after removal of the Softcup from the vagina); subsequent measurements were made 2 min apart for a total of 16 min.

Information about vaginal douching behavior and the use of oral or vaginal probiotics was not collected in this study.

### Analysis of cervicovaginal fluid samples

At the end of the ex vivo pH measurements, the Softcup was placed in a pre-weighed, sterile 50 mL polypropylene conical tube that was spun at 500 *g* for 1 min in a Centra-8 centrifuge (International Equipment Company, Needham MA) to recover the collected CVF. The Softcup was discarded, the conical tube reweighed, and the mass of CVF recovered calculated. A sterile cotton swab (Puritan Medical, Guildford ME) was dipped into the CVF and rolled out onto a glass microscope slide (Thermo Fisher Scientific) that was air-dried for later Gram staining and Nugent scoring according to a standard protocol [41].

The remaining CVF was diluted five-fold with autoclaved, UV-treated dH_2_O and spun at 12,000 *g* for 3 min in a 5424 Microcentrifuge (Eppendorf North America, Hauppauge NY). The supernatant was transferred to a 1.5 mL cryo-safe microtube (Sarstedt Inc., Newton NC) and stored at − 20 °C for up to 2 weeks. Supernatants were thawed and brought to room temperature before the lactate concentration was measured using the D-lactic acid/L-lactic acid Enzymatic BioAnalysis UV methods kit (R-Biopharm, Darmstadt Germany). The manufacturer’s supplied method (intended for use with a cuvette) was adapted for a 96 well microplate (Corning Inc.): the final reaction volume was reduced from 2.22 mL to 180 μL. Additionally, instead of assaying for D-lactate and then L-lactate sequentially in a single reaction mixture, replicate reaction mixtures were prepared from each supernatant so that D-lactate and L-lactate could be assayed at the same time. Absorbances were measured at 340 nm using a SpectraMax 190 spectrophotometer (Molecular Devices LLC., Sunnyvale CA). Absorbance changes were converted to concentrations using a standard curve derived from solutions of known lactate concentrations, prepared from sodium lactate to prevent inaccuracies that may arise due to dimerization of lactic acid that occurs when it is stored at high concentrations.

### Hydrogen ion and lactate production by vaginal lactobacilli in vitro

After withdrawing the supernatant (see Analysis of cervicovaginal fluid samples above), the whole bacterial pellet was resuspended in 50 mL of lactobacilli-selective MS culture medium [42] and incubated anaerobically at 37 °C. By definition, these low-Nugent score samples are composed predominantly of lactobacilli. The use of MS medium, together with the decrease in pH associated with the growth of lactobacilli, discourage the growth of non-lactobacilli bacteria. A clear predominance of lactobacilli morphotypes in each culture was confirmed by microscopic examination at every data collection. These ‘whole-pellet’ cultures were chosen in preference to monoclonal ‘pure’ cultures to capture the interstrain diversity of lactobacilli in each sample.

At *t* = 0 and every subsequent morning and evening for up to 5 days, each culture was inverted gently several times to resuspend the bacteria and a 2 mL aliquot was withdrawn. The pH of the sample was measured using the same model #A57184 electrode and A221 meter. Each aliquot was then spun at 12,000 g for 3 min; the supernatant was withdrawn, frozen, and its lactate concentration assayed as for the supernatant prepared from CVF (see Analysis of cervicovaginal fluid above). To examine the effect of pH on lactate production, the remaining culture was titrated with 1 N NaOH (J.T. Baker Chemicals, Phillipsburg NJ) to pH 7.0 (the starting pH of the MS culture medium) and incubated for a further 2 days before pH and lactate concentration were measured as on previous days.

### Statistical analysis

Results are reported as mean ± standard deviation. The difference between two means was tested using a two-tailed Student’s *t* test (comparisons were paired unless otherwise indicated); *p* values ≤0.05 were considered to be statistically significant. Statistical analysis was performed using PHStat2 version 3.0 (Microsoft Excel add-on).

## Abbreviations

CO2: Carbon dioxide
CVF: Cervicovaginal fluid
NaOH: Sodium hydroxide
